# Supplementary material for: Abscisic Acid and Cytokinins Are Not Involved in the Regulation of Stomatal Conductance of Scots Pine Saplings during Post-Drought Recovery
Source: Biomolecules. 2023 Mar 13;13(3):523. doi: 10.3390/biom13030523 (PMC10046708; doi:10.3390/biom13030523)
Supplement: Supplementary file 1 [file biomolecules-13-00523-s001.zip › biomolecules-2190076-supplementary.pdf]

Table S1. The contents (pmol/g DW) of abscisic acid, cytokinins and their metabolites, and CK/ABA ratios in needles of Scots pine plants in Experiment 1.

**Hormones**	**Day 0**		**Day 3**			**Day 14**		
	control	drought	control	drought	recovery	control	drought	recovery
				ABA and metabolites (pmol/g DW)				
ABA	301.0 ± 49.0	752.5 ± 73.7*	389.1 ± 23.1	1338.0 ± 142.2*	483.7 ± 34.2*	366.4 ± 54.3	2144.9 ± 191.2*	464.4 ± 67.6
ABA-Me	19.7 ± 2.9	52.8 ± 12.3*	28.8 ± 3.2	118.8 ± 26.1*	30.2 ± 3.4	33.4 ± 5.2	85.6 ± 23.4*	23.0 ± 4.6
ABA-GE	102784 ± 8480	125282 ± 10258	129730 ± 13900	186827 ± 13187*	122316 ± 10039	98030 ± 7921	88308 ± 12922	84177 ± 63661
PA	233.7 ± 16.9	177.0 ± 39.6	245.9 ± 36.5	220.5 ± 32.2	136.1 ± 11.3*	165.6 ± 15.5	363.9 ± 100.8	110.9 ± 18.2*
DPA	20.7 ± 4.2	12.9 ± 2.5	18.3 ± 3.9	15.7 ± 2.0	41.9 ± 8.4*	14.6 ± 1.7	57.0 ± 14.0*	30.3 ± 9.8
7OH-ABA	200.9 ± 23.0	772.2 ± 99.5*	225.4 ± 37.8	2254.9 ± 232.7*	1470.2 ± 313.6*	367.8 ± 69.4	5131.5 ± 1030.0*	458.4 ± 89.7
9OH-ABA	231.1 ± 26.0	140.2 ± 22.4	226.9 ± 33.7	240.3 ± 40.4	132.0 ± 15.3*	152.9 ± 14.0	335.9 ± 93.0	102.4 ± 16.8*
				Cytokinins and metabolites (pmol/g DW)				
*t*Z	0.83 ± 0.16	1.37 ± 0.11*	0.78 ± 0.10	1.53 ± 0.22*	0.96 ± 0.15	1.08 ± 0.10	3.79 ± 0.45*	1.50 ± 0.14*
DZ	0.97 ± 0.23	2.80 ± 0.54*	1.00 ± 0.20	2.25 ± 0.11*	3.54 ± 0.26*	1.05 ± 0.17	5.37 ± 0.31*	1.90 ± 0.35
iP	2.28 ± 0.27	1.83 ± 0.21	2.37 ± 0.28	3.75 ± 0.39*	3.09 ± 0.42	2.04 ± 0.41	2.23 ± 0.36	2.36 ± 0.32
*t*ZR	29.1 ± 3.0	37.3 ± 2.6	21.7 ± 1.3	31.0 ± 3.0*	37.9 ± 2.5*	26.5 ± 3.1	43.6 ± 4.4*	33.7 ± 2.8
DZR	15.7 ± 1.1	26.3 ± 2.1*	16.6 ± 1.6	18.6 ± 1.6	27.9 ± 4.9	16.4 ± 2.8	24.4 ± 3.3	23.1 ± 4.2
iPR	3.25 ± 0.59	5.95±1.17	4.09±0.62	8.17±0.95*	4.64±0.59	4.14±0.46	5.47±0.63	5.79±1.17
*c*ZR	11.0 ± 1.0	17.1 ± 1.6*	11.9 ± 2.0	16.7 ± 1.4	16.2 ± 1.3	11.6 ± 1.3	17.8 ± 2.9	14.6 ± 2.0
*t*ZOG	60.3 ± 5.3	62.6 ± 6.7	37.6 ± 3.8	42.0 ± 5.1	62.7 ± 10.3*	49.5 ± 4.0	93.4 ± 11.4*	64.8 ± 11.1
*t*ZROG	226.0 ± 18.5	222.2 ± 6.2	227.6 ± 23.8	238.1 ± 45.4	184.8 ± 20.2	250.8 ± 6.0	295.0 ± 22.5	322.3 ± 53.3
DZOG	107.6 ± 5.5	130.8 ± 15.8	114.6 ± 4.3	97.4 ± 18.2	134.3 ± 15.9	123.1 ± 12.5	159.6 ± 19.8	146.0 ± 25.3
DZROG	110.4 ± 18.0	102.8 ± 8.7	77.2 ± 5.6	108.6 ± 14.8	100.8 ± 13.9	95.7 ± 11.4	107.7 ± 14.1	129.4 ± 18.3
*c*ZOG	20.9 ± 5.2	18.3 ± 2.3	13.9 ± 2.9	16.9 ± 2.4	34.0 ± 9.7	61.8 ± 13.6	29.8 ± 7.1	31.3 ± 9.7
*c*ZROG	594.6 ± 82.0	658.0 ± 57.5	595.9 ± 35.2	611.5 ± 47.4	781.5 ± 51.8*	748.4 ± 34.0	695.0 ± 37.1	885.3 ± 59.0
*t*ZRMP	13.5 ± 1.7	15.2 ± 3.3	13.3 ± 1.9	16.4 ± 2.3	14.9 ± 2.0	16.3 ± 1.5	16.5 ± 2.4	18.8 ± 2.2
DZRMP	2.76 ± 0.63	21.4 ± 2.62*	4.32 ± 0.58	6.85 ± 0.90*	5.42 ± 1.01	5.17 ± 0.94	7.20 ± 0.57	8.13 ± 0.84*
iPRMP	18.2 ± 2.2	21.1 ± 3.6	15.4 ± 1.4	25.5 ± 2.0*	22.0 ± 3.3	21.9 ± 3.8	14.9 ± 2.3	25.6 ± 2.2
*c*ZRMP	17.8 ± 2.9	49.7 ± 8.5*	22.0 ± 3.4	43.3 ± 7.9	45.9 ± 9.3	25.2 ± 3.0	35.3 ± 3.3*	27.8 ± 2.5
				CK/ABA ratios				
*t*Z/ABA	0.0034 ± 0.0010	0.0019 ± 0.0002	0.0020 ± 0.0002	0.0012 ± 0.0002*	0.0020 ± 0.0003	0.0032 ± 0.0004	0.0018 ± 0.0002*	0.0039 ± 0.0010
DZ/ABA	0.0034 ± 0.0007	0.0036 ± 0.0005	0.0026 ± 0.0005	0.0018 ± 0.0002	0.0076 ± 0.0009*	0.0032 ± 0.0006	0.0026 ± 0.0001	0.0041 ± 0.0005
iP/ABA	0.0085 ± 0.0014	0.0025 ± 0.0002*	0.0063 ± 0.0009	0.0029 ± 0.0002*	0.0068 ± 0.0013	0.0057 ± 0.0010	0.0011 ± 0.0002*	0.0063 ± 0.0019

ABA, abscisic acid; ABA-Me, ABA-methyl ester; ABA-GE, ABA-glucosyl ester; PA, phaseic acid; DPA, dihydrophaseic acid; 7OH-ABA, 7-hydroxy-ABA; 9OH-ABA, 9-hydroxy-ABA; *t*Z, *trans*-zeatin; DZ, dihydrozeatin; iP, N^6-(Δ^2-isopentenyl)adenine; *t*ZR, *trans*-zeatin 9-riboside; DZR, dihydrozeatin 9-riboside; iPR, N^6-(Δ^2-isopentenyl)adenosine; *c*ZR, *cis*-zeatin 9-riboside; *t*ZOG, *trans*-zeatin O-glucoside; *t*ZROG, *trans*-zeatin 9-riboside O-glucoside; DZOG, dihydrozeatin O-glucoside; DZROG, dihydrozeatin 9-riboside O-glucoside; *c*ZOG, *cis*-zeatin O-glucoside; *c*ZROG, *cis*-zeatin 9-riboside O-glucoside; *t*ZRMP, *trans*-zeatin 9-riboside-5′-monophosphate; DZRMP, dihydrozeatin 9-riboside-5′-monophosphate; iPRMP, N^6-(Δ^2-isopentenyl)adenosine-5′-monophosphate; *c*ZRMP, *cis*-zeatin 9-riboside-5′-monophosphate. Pairwise comparisons of the means were performed between control and drought-stressed plants and control and recovering plants at each separate time point using Student's *t* test or Mann–Whitney rank sum test. Asterisks (*) denote significant differences at $p < 0.05$ between the control and experimental variants at each time point.

Table S2. Pearson correlations between contents of abscisic acid and their metabolites.

	**ABA-Me**	**ABA-GE**	**PA**	**DPA**	**7OH-ABA**	**9OH-ABA**
ABA	**0.66*** **	0.17	**0.53*** **	**0.38** **	**0.89*** **	**0.54*** **
ABA-Me		**0.37** **	0.23	-0.06	**0.64*** **	0.22
ABA-GE			0.01	-0.21	-0.04	0.11
PA				**0.35* **	**0.53*** **	**0.95*** **
DPA					**0.40** **	**0.34* **
7OH-ABA						**0.53*** **

* Pairs with $p < 0.050$; ** pairs with $p < 0.010$; *** pairs with $p < 0.001$. ABA, abscisic acid; ABA-Me, ABA-methyl ester; ABA-GE, ABA-glucosyl ester; PA, phaseic acid; DPA, dihydrophaseic acid; 7OH-ABA, 7-hydroxy-ABA; 9OH-ABA, 9-hydroxy-ABA.

Table S3. Pearson correlations between contents of cytokinins and their metabolites.

	Cytokinins and metabolites			
	*t*ZR	*t*Z	*t*ZOG	*t*ZROG
*t*ZRMP	0.17	0.03	-0.09	0.25
*t*ZR		**0.45** **	**0.44*** **	**0.31* **
*t*Z			**0.52*** **	**0.34* **
*t*ZOG				**0.43** **
	DZR	DZ	DZOG	DZROG
DZRMP	**0.36* **	**0.30* **	0.21	0.06
DZR		**0.50*** **	**0.60*** **	**0.42*** **
DZ			**0.44** **	0.16
DZOG				**0.56*** **
	*c*ZR	*c*ZOG	*c*ZROG	-
*c*ZRMP	0.28	0.09	-0.07	-
*c*ZR		-0.04	0.05	-
*c*ZOG			0.22	-
	iPR	iPR-MeS	iP	iP7G
iPRMP	**0.52*** **	-0.09	0.01	0.16
iPR		0.03	0.05	0.11
iPR-MeS			0.03	**0.32* **
iP				-0.27

* Pairs with $p < 0.050$; ** pairs with $p < 0.010$; *** pairs with $p < 0.001$. *t*Z, *trans*-zeatin; DZ, dihydrozeatin; iP, N^6-(Δ^2-isopentenyl)adenine; *t*ZR, *trans*-zeatin 9-riboside; DZR, dihydrozeatin 9-riboside; iPR, N^6-(Δ^2-isopentenyl)adenosine; *c*ZR, *cis*-zeatin 9-riboside; *t*ZOG, *trans*-zeatin O-glucoside; *t*ZROG, *trans*-zeatin 9-riboside O-glucoside; DZOG, dihydrozeatin O-glucoside; DZROG, dihydrozeatin 9-riboside O-glucoside; *c*ZOG, *cis*-zeatin O-glucoside; *c*ZROG, *cis*-zeatin 9-riboside O-glucoside; *t*ZRMP, *trans*-zeatin 9-riboside-5′-monophosphate; DZRMP, dihydrozeatin 9-riboside-5′-monophosphate; iPRMP, N^6-(Δ^2-isopentenyl)adenosine-5′-monophosphate; *c*ZRMP, *cis*-zeatin 9-riboside-5′-monophosphate; iP7G, isopentenyl adenine-7-glucoside; iPR-MeS, 2-methylthio isopentenyl adenosine.
